# PGBD5: a neural-specific intron-containing piggyBac transposase domesticated over 500 million years ago and conserved from cephalochordates to humans

**DOI:** 10.1186/1759-8753-4-23

**Published:** 2013-11-01

**Authors:** Thomas Pavelitz, Lucas T Gray, Stephanie L Padilla, Arnold D Bailey, Alan M Weiner

**Affiliations:** 1 Department of Biochemistry, School of Medicine, University of Washington, Seattle, WA 98195-7350, USA; 2 Howard Hughes Medical Institute, University of Washington, Seattle, WA 98195-7350, USA

**Keywords:** *Branchiostoma*, Central nervous system, Cephalochordate, Domestication, Granule cells PGBD5, PiggyBac

## Abstract

**Background:**

piggyBac domain (PGBD) transposons are found in organisms ranging from fungi to humans. Three domesticated piggyBac elements have been described. In the ciliates *Paramecium tetraurelia* and *Tetrahymena thermophila*, homologs known as piggyMacs excise internal eliminated sequences from germline micronuclear DNA during regeneration of the new somatic macronucleus. In primates, a PGBD3 element inserted into the Cockayne syndrome group B (CSB) gene over 43 Mya serves as an alternative 3′ terminal exon, enabling the CSB gene to generate both full length CSB and a conserved CSB-PGBD3 fusion protein that joins an N-terminal CSB domain to the C-terminal transposase domain.

**Results:**

We describe a fourth domesticated piggyBac element called PGBD5. We show that i) PGBD5 was first domesticated in the common ancestor of the cephalochordate *Branchiostoma floridae* (aka lancelet or amphioxus) and vertebrates, and is conserved in all vertebrates including lamprey but cannot be found in more basal urochordates, hemichordates, or echinoderms; ii) the lancelet, lamprey, and human PGBD5 genes are syntenic and orthologous; iii) no potentially mobile ancestral PGBD5 elements can be identified in other more deeply rooted organisms; iv) although derived from an IS4-related transposase of the RNase H clan, PGBD5 protein is unlikely to retain enzymatic activity because the catalytic DDD(D) motif is not conserved; v) PGBD5 is preferentially expressed in certain granule cell lineages of the brain and in the central nervous system based on available mouse and human *in situ* hybridization data, and the tissue-specificity of documented mammalian EST and mRNA clones; vi) the human PGBD5 promoter and gene region is rich in bound regulatory factors including the neuron-restrictive silencer factors NRSF/REST and CoREST, as well as SIN3, KAP1, STAT3, and CTCF; and vii) despite preferential localization within the nucleus, PGBD5 protein is unlikely to bind DNA or chromatin as neither DNase I digestion nor high salt extraction release PGBD5 from fractionated mouse brain nuclei.

**Conclusions:**

We speculate that the neural-specific PGBD5 transposase was domesticated >500 My after cephalochordates and vertebrates split from urochordates, and that PGBD5 may have played a role in the evolution of a primitive deuterostome neural network into a centralized nervous system.

## Background

piggyBac family transposons have been identified in fungi, protozoa, cnidarians, plants, insects, crustaceans, echinoderms, urochordates (aka tunicates), hemichordates (acorn worm), fish, amphibia, and mammals suggesting both horizontal transmission and occasional domestication [1]. For example, the domesticated piggyMac transposases are catalytically active and required for programmed genome rearrangements in the ciliates *Paramecium tetraurelia*[2] and *Tetrahymena thermophila*[3]. However, piggyBacs are not the only active domesticated transposases: the RAG1/2 recombinases of the human immune system are presumably descended from an ancient Transib transposase [4-7] and, most recently, the human THAP9 gene has been found to encode a catalytically active P-element DNA transposase of as yet unknown function [8]. Some domesticated transposons such as the centomere protein CENPB retain nuclear localization and specific DNA binding but have lost enzymatic function [9,10], whereas others such as SETMAR/Metnase [11,12] and the Cockayne syndrome Group B-PiggyBac domain 3 (CSB-PGBD3) fusion protein [13-15] retain site-specific DNA binding but gain new functions by fusion with upstream coding exons.

Most mammalian genomes contain only a handful of decayed piggyBac transposons. piggyBac transposons ceased activity over 35–40 Mya in the anthropoid primate lineage [16] and only somewhat more recently in the mouse lemur (prosimian) lineage [17]. The only exception known is the little brown bat, *Myotis lucifugus*, which contains thousands of active piggyBat elements [18,19]. Humans have 5 substantially complete piggyBac elements, designated PGBD1, 2, 3, 4, and 5. PGBD1, 2, and 3 have multiple coding exons, but in each case the piggyBac transposase-related sequence is encoded by a single uninterrupted 3′ terminal exon. Thus, PGBD1 and 2 may resemble the PGBD3 transposon in which the transposase ORF is flanked upstream by a 3′ splice site and downstream by a polyadenylation site [13,15]. As a result, insertion of PGBD3 into intron 5 of the CSB host gene enables the transposon to take advantage of the CSB promoter, using transposon-encoded alternative mRNA splicing and polyadenylation signals to express transposase as a CSB-PGBD3 fusion protein. The mouse genome lacks PGBD2, 3, and 4 homologs, but contains a modestly conserved PGBD1 suggesting introduction early in the mammalian radiation.

We now find that PGBD5, unlike other vertebrate piggyBacs, has been highly conserved in sequence and synteny from the primitive cephalochordate *Branchiostoma floridae* and the lamprey *Petromyzon marinus* (an agnathan or jawless fish) to humans. PGBD5, like other piggyBac elements, belongs to the RNase H clan of Pfam structures (pfam.sanger.ac.uk), and exhibits a highly significant match (1.7e-80) to the Hidden Markov Model for eubacterial and archaeal IS4 transposases [20]. Whereas PGBD3 has sustained only a single D to N mutation in the essential catalytic triad DDD(D) [13] and retains the ability to bind the upstream piggyBac terminal inverted repeat [15], PGBD5 lacks 3 of the 4 conserved catalytic piggyBac aspartates [21] and does not appear to bind either DNA or chromatin; moreover, in contrast to all other piggyBac elements except the ciliate piggyMacs [2,3], the PGBD5 transposase domain is encoded not by a single uninterrupted ORF but by 7 exons separated by long canonical introns most of which are conserved in position although intron loss and gain, or perhaps sliding, may also be involved. Taken together, the genomic data suggest that human PGBD5 is the most highly conserved piggyBac sequence known, and that it dates back over 500 My to the beginning of the chordate lineage.

An ancestral deuterostome is thought to have given rise to hemichordates and echinoderms through one line of descent and to chordates (including urochordates, cephalochordates, and vertebrates) through the other [22]. We show that PGBD5 is conserved from the primitive cephalochordate *Branchiostoma floridae* (aka lancelet or amphioxus) to humans, but appears to be absent in hemichordates, echinoderms, and urochordates. We also show that mouse PGBD5 is mainly nuclear and, consistent with publically available *in situ* hybridization and expression data, preferentially expressed in specific areas of the brain and central nervous system (CNS) that are enriched in granule cells. We also show that PGBD5 exon 1 is typically located far upstream from exons 2–7, and is embedded in a CpG island that is rich in bound neural transcriptional and regulatory factors. Finally, the PGBD5 transposase does not retain the catalytic DDD(D) motif found in active piggyBac elements, and is not released from nuclei by DNase I digestion or high salt extraction, suggesting that the transposase is not only inactive but fails to associate with either DNA or chromatin *in vivo*. Although many other scenarios can be imagined, we suggest, on the basis of these data, that PGBD5 may be expressed mainly, albeit not exclusively, in granule cells — an anatomically and functionally diverse population of small neurons, some of which are capable of adult neurogenesis [23-25].

## Results and discussion

### Assembly of the human PGBD5 gene

**Figure 1:**
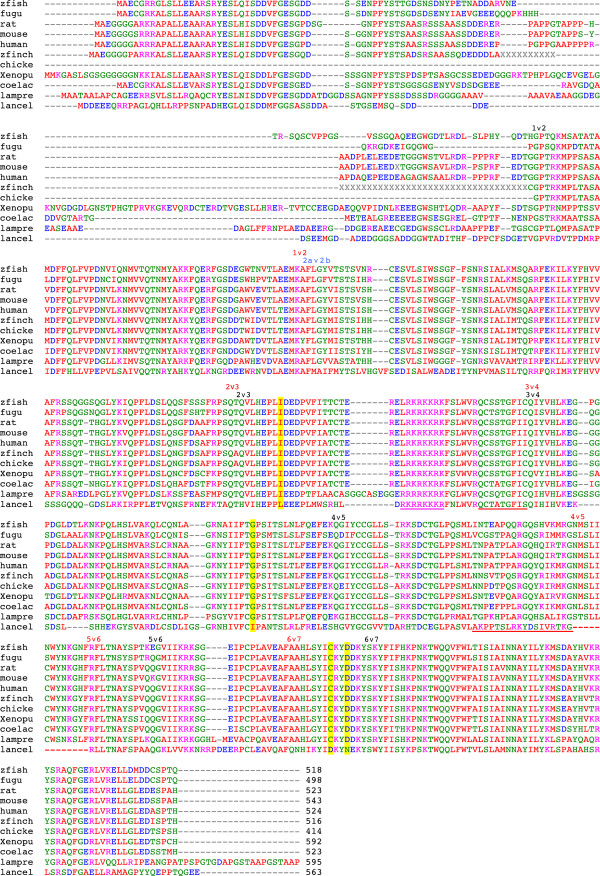
**Clustal alignment of representative PGBD5 orthologs including *Petromyzon marinus *(sea lamprey) and *Branchiostoma floridae *(lancelet or amphioxus).** For simplicity, many complete or partial vertebrate PGBD5s have been omitted from the alignment. The N-terminal motifs encoded by exon 1 are moderately conserved in all species including zebra finch, suggesting that chicken exon 1 lies within an unsequenced 770 bp gap located 29 kb upstream of exon 2 and immediately downstream of the sole substantial CpG island in the vicinity of the gene. Although human PGBD5 lacks 3 of the 4 catalytically active aspartates that are often conserved among diverse piggyBac elements, the positions of these four aspartates in a ClustalW alignment of piggyBac proteins most closely related to the active cabbage looper moth (*Trichoplusia ni*) transposase including human PGBD1, 2, 3, 4, and 5 [21] are highlighted in yellow. The Pfam homology with eubacterial and archeal IS4 transposases of the RNase H clan spans almost all of human PGBD5 exons 2–7 (residues 121–487). **Figure key:** black carets, vertebrate introns; blue caret, lamprey intron apparently orthologous to lancelet although shifted by 3 residues; red carets, lancelet introns; red underline, lancelet protein sequence derived from genomic tandem repeats (Additional file 1); red dashes, 13 residue deletion resulting from exclusion of predicted lancelet exon 5 which is embedded within the 108 bp genomic tandem repeats and would, if included, result in the 57 residue insertion; magenta underline, predicted nuclear localization signal not conserved in active Trichoplusia ni transposase; yellow highlight, position of four conserved, catalytic aspartates in active piggyBac transposases and homologs including human PGBD1, 2, 3, 4, and 5; gray XXX, regions of known length but undetermined sequence arbitrarily positioned in the clustal alignment; zfish, zfinch, xenopu, coelac, lampre, lancel are zebrafish, zebra finch, Xenopus tropicalis, coelacanth, lamprey, and lancelet respectively. Amino acid residues are colored according to the EBI Clustal convention for side chains (red, AVFPMILW; blue, DE; magenta, RK; green, STYHCNGQ; others, grey). To avoid prejudicial judgments regarding the relationship between highly divergent sequences, we refrained from assigning a similarity or homology score to each residue.

An initial BLAST search of the human genome hg18 build in 2007 using the complete PGBD3 transposon from intron 5 of the human CSB gene [13] identified PGBD5 as a distant homolog of PGBD3. Unlike most other piggyBac elements which appear to be previously or currently mobile and to lack introns within the transposase domain, PGBD5 is a single copy gene with multiple introns within the transposase domain and is highly conserved among other vertebrates in intron/exon structure and protein sequence. Intriguingly, the annotated human PGBD5 open reading frame (ORF) in both the UCSC and RefSeq gene tracks of the UCSC Genome Browser continued through the 5′ end of the first annotated exon, which was flanked by canonical 3′ and 5′ splice sites. We therefore searched for additional exons upstream. A single expressed sequence tag (EST) clone CX757968 was annotated as joining a remote 5′ exon in frame to the downstream ORF. A 68 kb intron separating exon 1 from exons 2–7 seemed improbable, especially when represented at the time by only a single EST; however, as illustrated in Figure 1 and discussed in detail below, the evidence for this remote exon 1 quickly became convincing: i) CX757968 was annotated as joining exons 1–3, but upon sequencing we discovered that this EST is in fact a partial 3′ mRNA spanning PGBD5 exons 1–7 (see Methods for details); ii) splicing of CX757968 exon 1 to PGBD5 exons 2–7 generates a continuous ORF in which exon 1 encodes protein motifs that are moderately conserved in the orthologous exon 1 of other vertebrate PGBD5 genes, as well as the more ancient lamprey and lancelet/amphioxus genes; iii) exon 1 lies within the major CpG island located in or near each of the orthologous vertebrate PGBD5 genes; iv) the CpG island is rich in occupied transcription factor (TF) binding sites including the Neural-Restrictive Silencing Factor/RE1-Silencing TF (NRSF/REST); v) the great majority of available vertebrate PGBD5 mRNAs and ESTs are derived from neural or pluripotent tissues; vi) PGBD5 is almost exclusively expressed in the brain, with preferential expression in certain granule cell lineages; and vii) the CpG island and/or nearby sequences exhibit histone modification patterns associated with poised or weak promoters in 9 non-neural cell lines.

### The search for more distantly related PGBD5 orthologs

**Figure 2:**
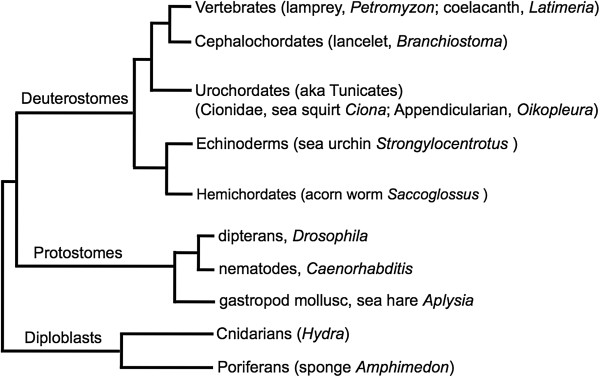
**Simplified phylogenetic tree of organisms examined.** PGBD5 homologs are found in cephalochordates and all vertebrates examined, but nowhere else. This cladogram does not imply either the timing or degree of evolutionary divergence.

As shown in Figure 1 (also see Methods and Additional file 1), we readily found orthologs of PGBD5 in the lamprey *Petromyzon marinus*, a jawless (agnathan) fish with a cartilaginous skeleton that is generally considered one of the most ancestral living vertebrates [26] and also in the lancelet *Branchiostoma floridae* (aka amphioxus), an even more primitive marine cephalochordate (Figure 2) with a notochord surmounted by a nerve chord leading to a single anterior eye.

*Saccoglossus kowalevichii* (acorn worm) is a direct developing hemichordate that can provide important clues regarding the origin of chordates (Figure 2). An ancestral deuterostome is thought to have given rise to hemichordates and echinoderms through one line of descent, and to chordates, including the urochordates (aka tunicates), cephalochordates, and vertebrates, through another; moreover, a strong case can be made that similar genetic pathways are used to build the hemichordate and chordate body plans [22] and that hemichordates and chordates use similar genes to define anteroposterior neuroanatomy despite the evolutionary leap from a diffuse nerve network in hemichordates to the centralized chordate nervous system [27,28].

A tBLASTn search of the NCBI genomic and EST databases for the acorn worm using the complete lancelet PGBD5 protein sequence as query generated only a few weak, fragmentary hits, although ESTs from three different developmental stages were included among the sequence libraries (mixed blastula and gastrula, mixed late gastrula and neurula, and early juveniles) [29]. In contrast, BLASTing the Skow_1.1 draft assembly of the *S. kowalevichii* genome (hgsc.bcm.edu/content/acorn-worm-genome-project) revealed two groups of distant piggyBac homologs, 2 on contigs 47239 and 124986, and 3 on contigs 10046, 73983, and 91916; however, neither group was more closely related to human PGBD5 than PGBD3, and all 5 homologs had continuous intronless ORFs unlike PGBD5. Although there are precedents for functional, developmentally regulated retrogenes [30], exceedingly weak homology and the absence of introns suggest that acorn worm lacks a PGBD5 ortholog.

Similarly, only distant intronless piggyBac ORFs were found in three representative deuterostomes, the echinoderm *Strongylocentrotus purpuratus* (sea urchin), and the urochordates *Ciona intestinalis* (sea squirt) and *Oikopleura dioica* (an appendicularian). We note, however, that an argument can be made on the basis of whole genome comparisons that chordates are not necessarily monophyletic and that *Oikopleura*, instead of cephalochordates, may be the closest living relative of vertebrates [31]. No PGBD5 orthologs or homologs were found in the nematode *C. elegans*, the dipteran *D. melanogaster*, the cnidarian *Hydra magnipapillata*, the gastropod mollusc *Aplysia californica* (sea hare), or the poriferan *Amphimedon queenslandica* (marine sponge)*.* We conclude that PGBD5 is likely to have originated in the chordate lineage and not in the ancestral deuterostome that gave rise to hemichordates and chordates (Figure 2).

### Orthology of lancelet and lamprey PGBD5s with vertebrate PGBD5s

**Figure 3:**
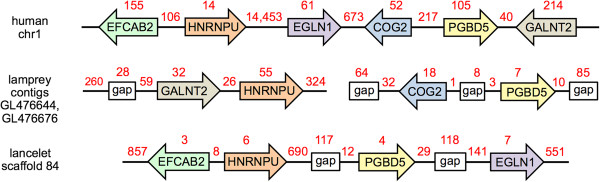
**Lancelet, lamprey, and human PGBD5 are orthologous.** Only shared syntenic genes are shown. The schematic is not drawn to scale, introns are not shown, genomic gaps are unsequenced, and distances are indicated in kb (red). The PGBD5 orthologs are oriented for clarity; the order and orientation of the two lamprey scaffolds is arbitrary. The 5′ and 3′ ends of lamprey GALNT2 are joined in the UCSC browser based on homology to the 5′ and 3′ ends of vertebrate GALNT2. Although >500 families of transposable elements constitute approximately 30% of the lancelet genome [32], no other PGBD5 homologs or fragments are found in the lancelet v2.0 draft genome; very similar PGBD5 sequences are present in both scaffolds 83 and 84 of the v1.0 draft genome (genome.jgi-psf.org/Brafl1/Brafl1.home.html), but PGBD5 appears only once in the v2.0 draft genome (downloadable from the UCSC browser at hgdownload.cse.ucsc.edu/gbdb/braFlo2/) in a sequence context most closely resembling scaffold 84 (Additional file 1).

Orthologous genes from lancelet scaffolds tend to be concentrated in specific regions of human chromosomes, consistent with conservation of gene linkage between lancelet and vertebrates on a whole chromosome scale (macro-synteny) but limited conservation of local gene order (micro-synteny) [32]. A careful comparison of the micro and macro environment of the lancelet, lamprey, and human PGBD5 genes reveals substantial conservation at both levels, providing strong evidence that the lancelet, lamprey, and vertebrate PGBD5s are orthologous (Figure 3). Specifically, i) the synteny and orientation of 3 genes neighboring lancelet PGBD5 appears to be conserved in humans, and the change in gene order from EFCAB2-HNRNPU-PGBD5-EGLN1 in lancelet to EFCAB2-HNRNPU-EGLN1-PGBD5 in humans is consistent with both macro- and micro-synteny; ii) PGBD5 and COG2 are divergently transcribed in lamprey and human, but not closely linked in lancelet, suggesting that this gene order may have been established at the base of the vertebrate lineage; iii) GALNT2 and HNRNPU are syntenic in lamprey and human, consistent with macro-synteny; and iv) the lancelet, lamprey, and human PGBD5s each appear to be single copy, highly homologous to each other, but only distantly related to other piggyBac families and primate PGBD1, 2, 3, and 4 [1]. We conclude that lancelet, lamprey, and human PGBD5 are orthologs.

We were unable to trace the lancelet PGBD5 gene neighborhood further back in time to a more basal chordate. Homologs of EFCAB2, HNRNPU, EGLN1/2, and GALNT2 in the JGI v2 draft genome of the tunicate *Ciona intestinalis* are located on different scaffolds, and no COG2 homolog could be found by a BLAT search with human COG2 (genome.jgi-psf.org/Cioin2/Cioin2.home.html). In another tunicate, the appendicularian *Oikopleura dioica*, only a GALNT2 homolog could be found among the existing scaffolds (http://www.genoscope.cns.fr/externe/GenomeBrowser/Oikopleura/). Nor could any PGBD5 homologs be found in either the Ciona or Oikopleura draft genomes. Thus, in the absence of flanking direct repeats or terminal inverted repeats in extant PGBD5s, we do not know whether the ancestral PGBD5 was a transposable element like most other piggyBacs, or even a cellular gene; whether and when it transposed or recombined into the neighborhood of EFCAB2, HNRNPU, and EGLN1/2; whether it had introns before arriving in the neighborhood, gained them once there, or has both gained and lost introns as demonstrated for chordate GIN1 and GIN2 integrase-related proteins [33]; whether it acquired a new, possibly neural-specific promoter and/or 5′ exon upon transposition or recombination, or continued to use an internal promoter as observed for the *Trichoplusia ni* piggyBac element in both insect [34] and mammalian cells [35]; and whether any ancestral PGBD5s have survived or are now all extinct.

Consistent with PGBD5 orthology, all vertebrates from lamprey to human share the same six introns (Figure 1, black carets). A seventh lamprey intron (blue caret) located in the middle of vertebrate exon 2 is shared with lancelet (red caret) and (despite a 3 amino acid residue shift between lancelet and lamprey) may be an orthologous intron that was lost in the last common ancestor of lamprey and true vertebrates. Although lancelet lacks vertebrate intron 1, it shares vertebrate introns 2 and 3; vertebrate introns 4, 5, and 6 are shifted by 11 to 42 amino acid residues relative to lancelet introns 4, 5, and 6, most probably reflecting a combination of intron loss and gain, rather than intron sliding which appears to be restricted to one or a few nucleotides [36]. We were unable to trace any PGBD5 sequences or intron positions further back than lancelet (Additional file 2).

We wish to stress that PGBD5 domestication (assuming PGBD5 descent from a transposable element) could have been a multistep process in which PGBD5 was initially selected or fixed for different reasons from those for which the gene is retained today. Indeed, PGBD5 could be retained for different reasons in lancelet and/or lamprey than in higher vertebrates which exhibit significantly greater PGBD5 sequence conservation (Figure 1, and Additional file 3). A similar scenario could hold for the SETMAR/Metnase fusion protein which evolved stepwise over a period of 18 My [11,12]. Orthology can define a line of descent, but not necessarily related gene functions, regulation, or selection.

### PGBD5 is primarily if not exclusively expressed in brain and CNS

**Figure 4:**
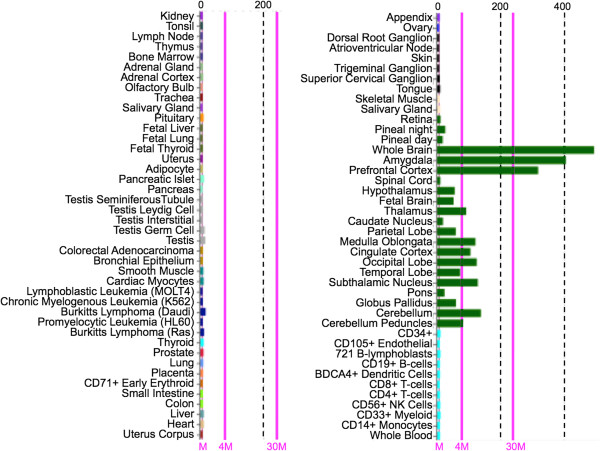
**PGBD5 is primarily if not exclusively expressed in brain and CNS.** Affymetrix expression profiles for 79 human tissues and cell lines as described [37] on the BioGPS Gene portal (biogps.org/) indicate that PGBD5 is strongly expressed in brain, and very weakly expressed in spinal cord as well as 4 different leukemia and lymphoma cell lines but not in normal B- and T-cells. Vertical gray dashed lines, levels of expression; vertical magenta solid lines, mean, 4× mean, and 30× mean expression.

The majority of available vertebrate PGBD5 mRNAs and ESTs are derived from embryonic, fetal, or adult brain (Additional file 4), consistent with Affymetrix expression profiles for 79 human tissues in the BioGPS gene portal (biogps.org) [37] indicating that PGBD5 is strongly expressed in brain, very weakly expressed in spinal cord, and also weakly expressed in 4 different leukemias and lymphomas (K562 chronic myelogenous leukemia, HL60 promyelocytic leukemia, and both Daudi and Raji Burkitt’s lymphoma) but not in a variety of normal B- and T-cells (Figure 4).

**Figure 5:**
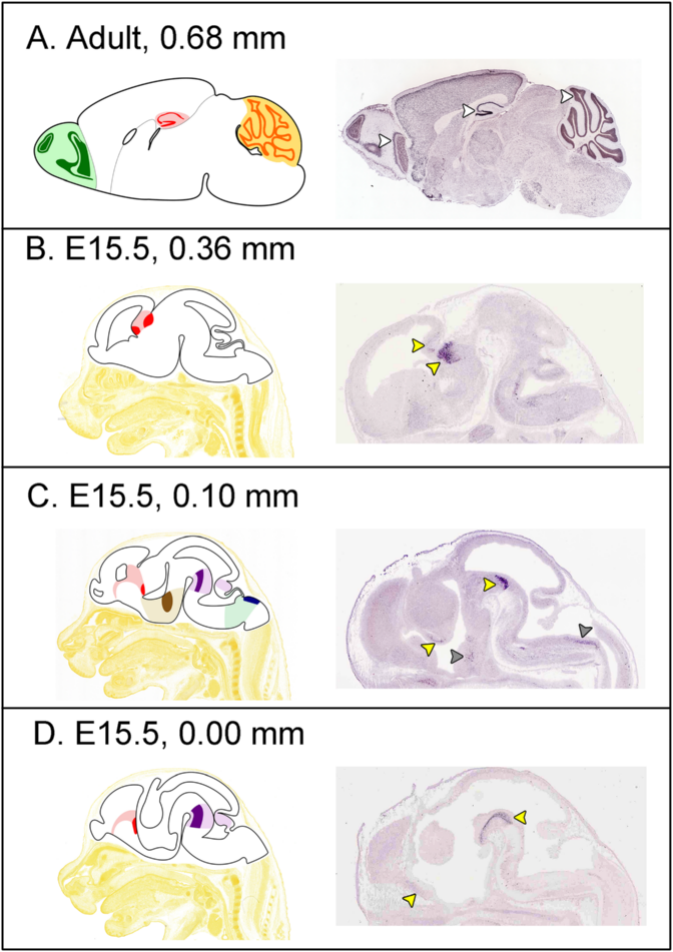
**PGBD5 expression in the adult and developing mouse brain.** Panels **A-D** illustrate expression of PGBD5 in sagittal brain sections (right) and a corresponding view from the Allen Institute Brain Atlas (left). The age of the mouse and the sagittal position relative to midline (0.00 mm) are indicated within each panel. The *in situ* hybridization stains are from public expression databases including The Allen Institute for Brain Science [39] for the adult mouse and Max Planck’s Genepaint [38] for embryos. The atlas images are color coded: olfactory bulb (green); cerebellum (orange); medial pallium (red); hypothalamus (brown); prepontine hindbrain (purple); and medullary hindbrain (blue). PGBD5 expression is restricted to a subset of cells within each nucleus denoted by a more saturated color. For example, PGBD5 expression in the cerebellar granule cell layer is indicated by a darker orange than the surrounding cerebellar cortex. The Max Planck and Allen Institute *in situ* hybridization probes both span exons 2–7, and are nearly identical; the absence of exon 1 is unlikely to affect the *in situ* patterns because there is no evidence in either the ENCODE (Additional file 5) or Chromatin State Segmentation data (Figure 6) of a functional promoter between exons 1 and 2.

To localize PGBD5 expression more precisely within the brain and CNS, we examined public *in situ* hybridization databases including Max Planck’s Genepaint [38] and the Allen Brain Atlas [39]. Consistent with the BioGPS dataset, the PGBD5 transcript is expressed in restricted regions of the murine CNS. Over the course of development, PGBD5 is expressed in immature cells of the medial pallium and prepontine isthmus which give rise to the hippocampus and cerebellum, respectively (Figure 5, yellow arrowheads). In midline sagittal sections of the embryo, PGBD5 expression can be seen in aspects of the presumptive hypothalamus and medulla (Figure 5, grey arrowheads). In the adult mouse brain, PGBD5 is predominantly expressed in granule cells, including those of the olfactory bulb, hippocampus and cerebellum (Figure 5, white arrowheads). Although embryonic PGBD5 expression in the medial pallium and prepontine isthmus apparently persists in the adult hippocampus and cerebellum, other embryonic sites of PGBD5 expression are lost in the adult including the hypothalamus and medulla.

Interestingly, PGBD5 is expressed in the granule layer of the olfactory bulb and dentate gyrus in the adult brain. Cells in both these regions are a mixture of mature and newborn granule neurons. In rodents, almost all neurogenesis occurs during embryogenesis and early perinatal development; however, the subventricular zone surrounding the lateral ventricles and the subgranular zone of the dentate gyrus are adult neurogenic niches, containing stem cells that give rise to adult-born granule cells in the olfactory bulb and dentate gyrus, respectively (for review see [24]). In the olfactory bulb, mature granule cells function as inhibitory interneurons that are thought to mediate contrast between odor stimuli [40] whereas adult-born granule cells in this region are induced by olfactory sensory inputs and may be involved in odor memory (for review see [41]). In the hippocampal dentate gyrus, mature granule cells mediate pattern completion during memory formation whereas adult-born granule cells are believed to facilitate pattern separation [42]. Although PGBD5 expression is seen in granule layers of the olfactory bulb and dentate gyrus by *in situ* hybridization, double labeling would be required to determine whether PGBD5 is expressed by mature or adult-born granule cells. Nevertheless, because PGBD5 continues to be expressed as the medial pallium develops into the mature hippocampus, we suspect that PGBD5-expressing granule neurons of the dentate gyrus are resident cells born during embryogenesis.

Using the Allen Institute’s microarray database, we also screened for PGBD5 expression in the adult human brain. As in murine brain, PGBD5 is expressed throughout the hippocampal formation, and in the pontine and aspects of the medulla including the raphe and arcuate. However, little or no PGBD5 expression was detected in human cerebellum by microarray analysis compared to mouse cerebellum by *in situ* hybridization.

### The human PGBD5 promoter is embedded in a prominent CpG island and binds the neuron-restrictive silencer factors NRSF/REST and CoREST

**Figure 6:**
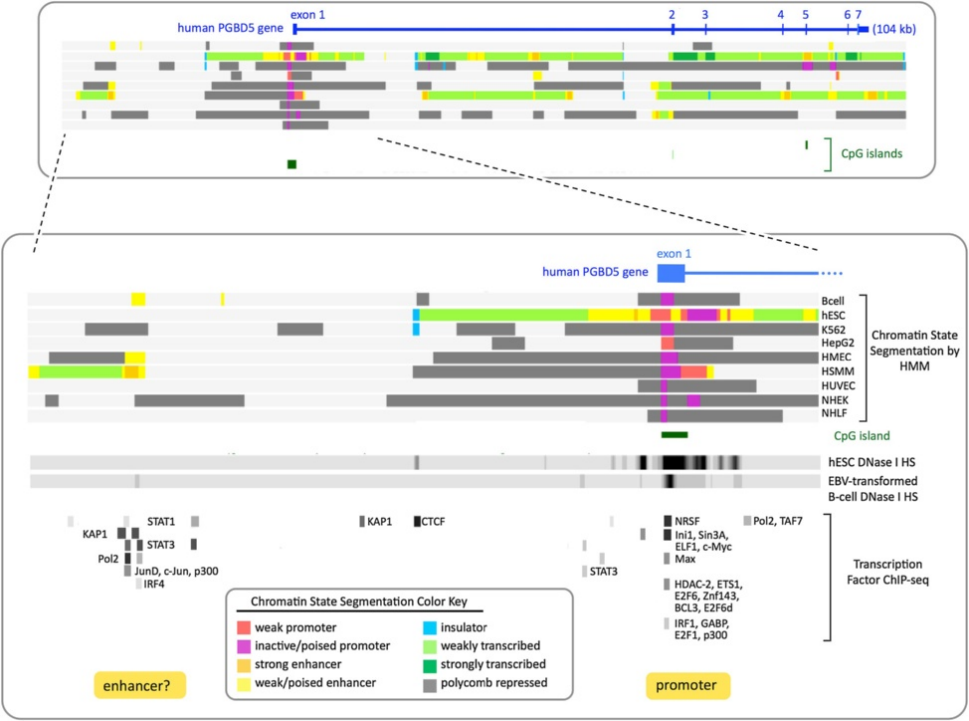
**PGBD5 chromatin structure confirms location of promoter and transcription start site. (Upper panel)** Structure of the human PGBD5 gene (top), chromatin state segmentation data from 9 non-neuronal cell lines (middle), and CpG islands (bottom). **(Lower panel)** An expanded view of the PGBD5 promoter and presumptive enhancer region. Chromatin state segmentation data are from the same 9 human cell types: GM12878 (EBV-transformed B-lymphocytes), H1-hESC (embryonic stem cells), K562 (chronic myelogenous leukemia), HepG2 (hepatocellular carcinoma), HMEC (mammary epithelial cells), HSMM (skeletal muscle myoblasts), HUVEC (umbilical vein endothelial cells), NHEK (epidermal keratinocytes), and NHLF (lung fibroblasts). The DNase I hypersensitive sites are from H1-hESC and GM12878. The TF ChIP-seq data are a composite of 24 neural and non-neural cell lines including H1-hESC, A549 (adenocarcinomic alveolar basal epithelial cells), BE2_C (brain neuroblastoma), HEK293 (embryonic kidney), HeLa (cervical carcinoma), HUVEC, Jurkat (T-lymphocyte), K562, HepG2, NB4 (acute promyelocytic leukemia), PANC-1 (pancreatic carcinoma), PFSK-1, SK-N-MC (neuronal epithelioma), SK-N-SH_RA (neuroblastoma differentiated with retinoic acid), and U87 (primary glioblastoma), and 10 EBV-transformed B-lymphocytes from various ethnic backgrounds. For a color key to the chromatin state segmentation data, see inset **(lower panel)**. For CpG islands, darker green indicates higher CpG density. For DNase I hypersensitive sites, stronger signals indicate higher sensitivity. For TF ChIP-seq data, darker gray reflects higher occupancy. All data is publicly available as UCSC Genome Browser tracks: Open Chromatin by DNase I HS (ENCODE/Duke University); TF ChIP-seq (ENCODE/Broad Institute/HudsonAlpha Institute/Stanford/Duke/University of Washington); and chromatin state segmentation using a Hidden Markov Model to identify genome-wide patterns in ChIP-seq data for histone methylations and acetylations, histone variant H2AZ, RNAP II, and CTCF (ChromHMM from ENCODE/Broad Institute). Only 8 of the 15 distinguishable chromatin states [48,49] are seen in this particular genomic interval of the 9 cell lines. Figure adapted from visualizations of current ENCODE databases available on the UCSC Genome Browser for hg19.

Human PGBD5 exon 1 is phylogenetically conserved from branchiostomes to vertebrates (Figure 1), co-localizes with the single most prominent CpG island in the entire PGBD5 gene region, and is located 68 kb upstream of PGBD5 exons 2–7 encoding the piggyBac transposase domain (Figure 6 and Additional file 5). This CpG island is likely to harbor the PGBD5 promoter because i) the island overlaps the single strongest cluster of DNase I hypersensitive sites (ENCODE/Duke) that are diagnostic of open chromatin; ii) the island also overlaps the only strong chromatin marks in the Chromatin State Segmentation database (ENCODE/Broad) that are diagnostic of weak and/or poised promoters; and iii) the most highly occupied TF binding sites (ENCODE/Broad/HudsonAlpha/ Stanford/Duke/University of Washington) either overlap the island or cluster 24 kb upstream in the presumptive enhancer.

Consistent with BioGPS analysis (Figure 4), a majority of the 62 non-neural cell lines used for acquisition of the ENCODE TF ChIP-seq data (Figure 6 and Additional file 5) exhibit a single highly occupied site in the PGBD5 promoter for NRSF/REST, a factor which serves as a scaffold for assembly of many other proteins that can repress neural genes in non-neural cells [43]. For example, the N-terminal domain of NRSF/REST binds the SIN3A co-repressor, which in turn binds the repressive HDAC1/2 (histone deacetylases 1 and 2) and STAT3, thereby acting as a context-dependent ISGF3/STAT3 transcriptional switch [44]; the C-terminal domain of NRSF/REST binds the CoREST co-repressor which not only binds HDAC1/2, but also the repressive DNMT1 (DNA methyltransferase 1), histone K4 demethylase, and histone K9 methyltransferase, thereby regulating gene networks that control neural stem cell fate decisions [45]; and the internal domain of NRSF/REST binds a family of DNA motifs that regulate neuronal gene networks [46]. Thus, binding of NRSF/REST to the CpG island in non-neural cells is likely to explain not only colocalization of HDAC2 but also the Ini1, BRG1, and BAF155 components of the SWI/SNF chromatin remodeling complex required for repression of neuronal genes by the C-terminal CoREST corepressor [47].

Similarly, although none of the 9 cell lines used to build the Hidden Markov Model for chromatin state segmentation is neuronal [48,49], all 9 lines display chromatin marks that are diagnostic of weak and poised promoters overlapping both the CpG island and exon 1 (Figure 6). Indeed, as might be expected, totipotent human H1 embryonic stem cells exhibit by far the most dramatic DNase I hypersensitivity indicative of open chromatin and the broadest chromatin state segmentation profile indicative of potential promoter activity.

NRSF/REST is unlikely to be the only repressor of PGBD5 in non-neural cells. Unexpectedly, loss of functional NRSF/REST during mouse and chicken development de-represses only a few neural genes in non-neural tissues, providing strong evidence for redundant repressors and/or the absence of neural activators in non-neural cells [50]. Consistent with this view, the ability of NRSF/REST to serve as a tumor suppressor in colorectal cancer [51] suggests that inappropriate expression of neural genes in non-neural tissues can have adverse consequences.

### PGBD5 is nuclear in mouse brain but not bound to chromatin or DNA

Although a domesticated transposase might be expected to function within the nucleus, domestication and exaptation are full of surprises [52]. Vertebrate PGBD5s do indeed appear to have a strong candidate nuclear localization signal (NLS) with the sequence LRKRKKRKF, and the lamprey (ERRRRKKRKF) and lancelet (DRKRRKKKRF) sequences differ only slightly (Figure 1, magenta underline); however, this candidate NLS sequence is unique to the PGBD5 family and is located in the middle of the conserved transposase core, whereas the experimentally confirmed bipartite NLS in the cabbage looper moth transposase is C-terminally located [53]. Moreover, NLS sequences are typically diverse, whereas the candidate PGBD5 NLS is nearly invariant, suggesting that it may not be an NLS or is highly conserved for unrelated structural or functional reasons.

**Figure 7:**
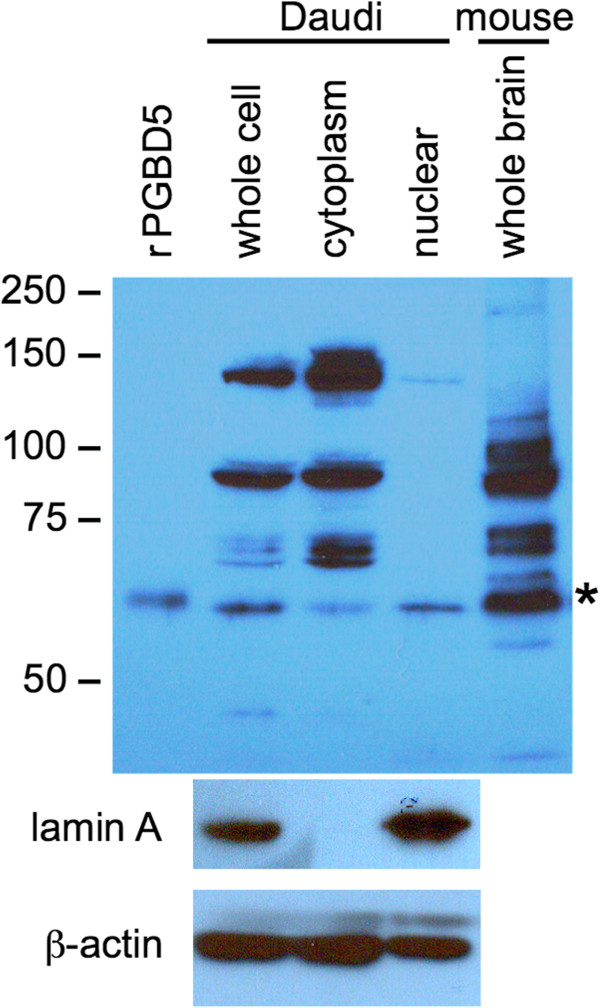
**PGBD5 partitions between nucleus and cytoplasm in the Daudi Burkitt’s lymphoma B-cell line.** Nuclear and cytoplasmic fractions were prepared as described in Methods. **(Upper panel)** Whole cell, cytoplasmic, and nuclear fractions of 2×10^5^ cells/lane were resolved by 7% SDS-PAGE, blotted, and probed with anti-rPGBD5 antibody. Mouse whole brain extract and human rPGBD5 served as controls. PGBD5 is indicated by an asterisk. Immunoreactive proteins larger than PGBD5 do not immunoprecipitate with anti-rPGBD5 antibody (compare with Figure 8). These proteins may cross-react weakly with antibody after denaturation and immobilization on the membrane, just as the antibody cross-reacts weakly with the hexahistidine tag but not the HA tag on western blotting (data not shown). **(Lower panels)** Daudi whole cell, cytoplasmic, and nuclear fractions were also probed for lamin A and β-actin to confirm successful partition.

**Figure 8:**
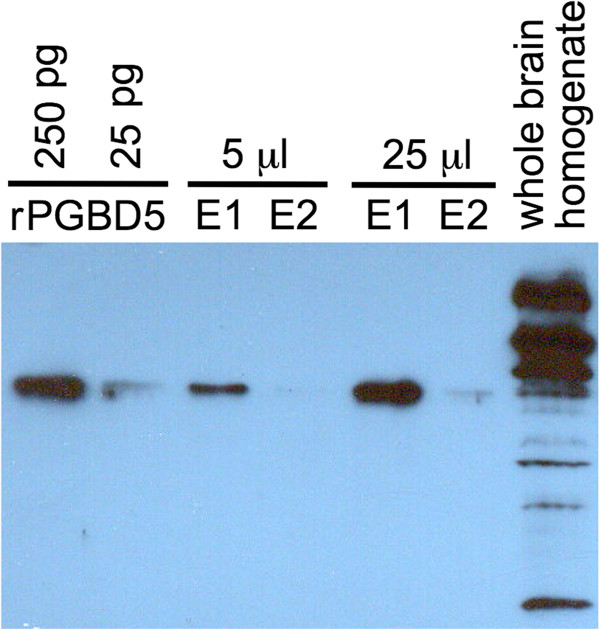
**Immunoprecipitation of endogenous PGBD5 from mouse brain.** Mouse whole brain homogenate (5 or 25 μL) was immunoprecipitated with anti-rPGBD5 antibody. The proteins were resolved by SDS-PAGE, blotted, and probed with the same anti-rPGBD5 antibody as in Figure 7. rPGBD5, recombinant PGBD5; E1 and E2, first and second SDS elutions from the beads. Note that proteins larger than PGBD5 are present in whole brain homogenate **(rightmost lane)** but do not immunoprecipitate (lanes E1 and E2).

Given strong if not exclusive expression of PGBD5 in brain and CNS (Figures 4 and 5), as well as unexpected expression in B- and T-cell tumors (Figure 4), we initially decided to examine the subcellular localization of PGBD5 in a panel of pediatric brain tumor cell lines [54]. However, our recombinant PGBD5 (rPGBD5) antibody did not immunoprecipitate any protein of the expected size (about 59 kDa) although several prominent larger bands were visible on western blots (data not shown). In contrast, the rPGBD5 antibody cleanly immunoprecipitated a protein of about 59 kDa from the Daudi Burkitt’s lymphoma B-cell line and also recognized this protein on western blots along with an array of large prominent bands resembling those seen with the pediatric brain tumors (data not shown). These large proteins do not immunoprecipitate with the rPGBD5 antibody (Figures 7 and 8), and cross-react weakly only after denaturation and immobilization on the membrane.

Although PGBD5 expression in leukemias and lymphomas (Figure 4) may be a fortuitous consequence of cell transformation, the convenience of working in an established cell line persuaded us to ask whether PGBD5 is nuclear or cytoplasmic in Daudi cells. Growing cells were washed and the plasma membrane disrupted by 0.1% NP40. Cytoplasmic and nuclear fractions were separated by centrifugation, the proteins resolved by SDS-PAGE, and PGBD5 assayed by western blotting with anti-rPGBD5 antibody (Figure 7). PGBD5 partitioned about equally to cytoplasmic and nuclear fractions whereas the large cross-reacting bands partitioned almost exclusively to the cytoplasm, suggesting that these may be abundant cytoskeletal proteins.

We ultimately decided to pursue PGBD5 subcellular localization *in vivo* using mouse whole brain homogenates because the mouse and human *in situ* hybridization data indicated expression of PGBD5 in only certain brain cell types (Figures 4 and 5). A BALBc mouse was euthanized, the brain including meninges removed and minced, and the tissue disrupted by Dounce homogenization with the loose and tight pestles in buffer containing protease inhibitors. The resulting homogenate was sonicated to open all cells, and then used without fractionation for immunoprecipitation and western blots. As expected, our polyclonal antibody against rPGBD5 cross-reacted with the nearly identical mouse PGBD5 (Figure 1), immunoprecipitating a single band from mouse brain that was close in size to rPGBD5 (Figure 8). As also observed for Daudi (Figure 7), mouse brain contains proteins that are larger than PGBD5 and cross-react on westerns but do not immunoprecipitate.

**Figure 9:**
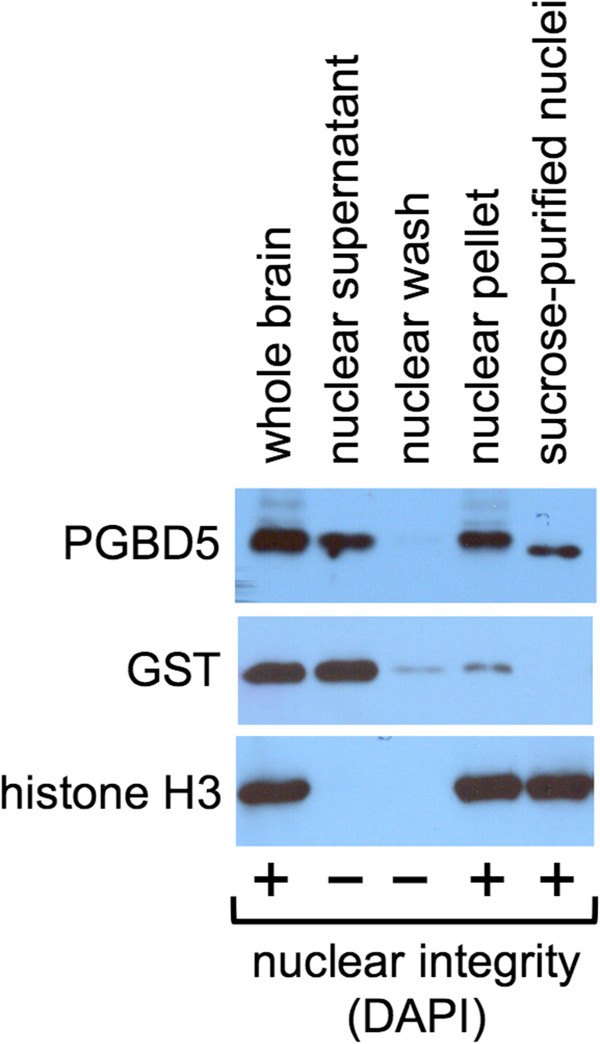
**PGBD5 partition in mouse brain.** Whole frozen mouse brains were thawed, Dounced with a tight pestle, and partitioned as described in Methods. Crude nuclei were pelleted through a sucrose cushion to remove debris and residual cytoplasm. Aliquots corresponding to equal cell equivalents were assayed by western blotting for PGBD5, glutathione-S-transferase (cytoplasmic marker), and histone H3 (nuclear marker). Nuclear integrity was determined visually by DAPI staining. As in Figure 8, PGBD5 partitioned about equally between nucleus and cytoplasm.

To date, all domesticated transposases of known or suspected function appear to be involved in nuclear DNA or chromatin transactions including SETMAR/Metnase [11,55,56], RAG1/2 [4-7], piggyMac [2,3], piggyBat [18,19], THAP9 [8], the TF ZBED6 [57], the major mammalian centromere protein CENP-B [9,10], and the CSB-PGBD3 fusion protein [13-15,58]. We therefore asked whether mouse brain PGBD5 was also nuclear and associated with chromatin or DNA. A frozen whole mouse brain (PelFreez) was crushed in liquid nitrogen, and then homogenized with the loose pestle to disrupt cells while leaving nuclei intact as judged by DAPI staining. Cytoplasmic and nuclear fractions were separated by centrifugation and assayed by western blotting for PGBD5, using histone H3 and glutathione-S-transferase to confirm successful cell fractionation (Figure 9). PGBD5 partitioned about equally into the cytoplasmic and nuclear fractions, but the crude nuclear pellet was heavily contaminated with insoluble brain matter. To separate intact nuclei from lighter membranous material, the crude nuclear pellet was centrifuged through a 1.8-M sucrose cushion. PGBD5 again partitioned with histone H3 (Figure 9, rightmost lane) confirming nuclear localization.

**Figure 10:**
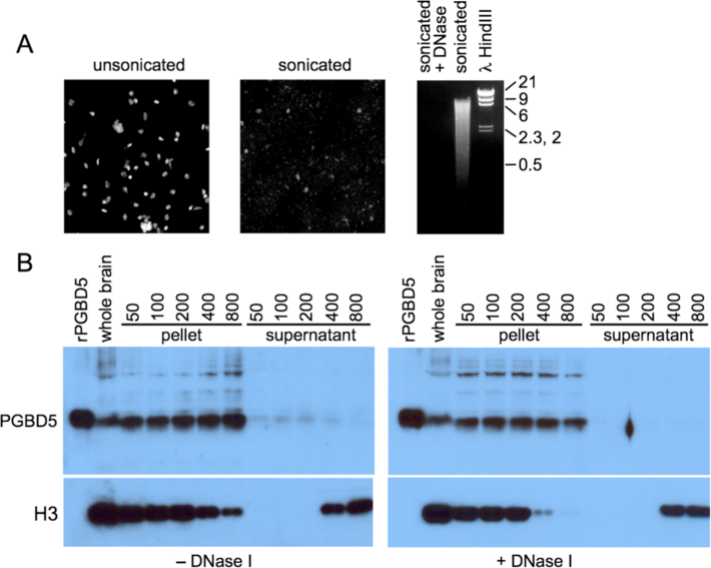
**Salt extraction of sonicated crude nuclei from mouse brain. (A)** Crude nuclei from mouse whole brain were lysed and chromatin sheared by sonication. Nuclear lysis was assayed by DAPI staining before and after sonication **(top left panel)**. Sonication was monitored by agarose gel electrophoresis with and without DNase I digestion **(top right panel)**. **(B)** Sonicated nuclei were extracted with NaCl at the indicated concentrations (mM) with or without prior DNase I digestion. Supernatant and pellet fractions were separated by centrifugation, resolved by SDS-PAGE, and assayed by western blotting for PGBD5 **(top panels)** or histone H3 **(bottom panels)**.

Lastly, we asked whether nuclear PGBD5 could be released by increasing salt concentrations as is the case for non-histone chromosomal proteins and histones. Aliquots of the crude nuclear pellet were extracted with NaCl concentrations from 50 to 800 mM, with and without prior DNase I digestion (Figure 10A). After re-pelleting the nuclei, supernatant and pellet fractions were assayed for PGBD5 by SDS-PAGE and western blotting. PGBD5 was not released by complete DNase I digestion or by NaCl concentrations as high as 800 mM which efficiently extract core histone H3 (Figure 10B, compare right and left panels). These results suggest that PGBD5 is a structural component of the nucleus, perhaps bound to the nuclear matrix or lamina, but do not rule out a weaker or reversible association with chromatin or DNA.

## Conclusions

We do not know why the intron-containing PGBD5 element first appears in cephalochordates like the lancelet, which has only minimal sensory awareness of light, touch, vibration, and may sense pheromones, but lacks a neural crest, most placodes, and the organizer dividing midbrain and hindbrain [28]. Nor do we know why the ur-PGBD5 element was lost from (or failed to invade) more primitive urochordate and deuterostome lineages. We also cannot explain why a domesticated transposase that presumably once catalyzed DNA transactions would no longer bind DNA or chromatin yet still localize to the nucleus. Nonetheless, although the potential roles of PGBD5 in chordate brain development, neural cell differentiation, and evolution remain a mystery, it is unlikely to be a coincidence that PGBD5, arguably the most unusual and highly conserved member of the large piggyBac superfamily, first arose in the earliest basal chordate with a primitive CNS.

A major question regarding the origin of our own CNS is whether the chordate dorsal CNS arose from a dorsal centralized, ventral centralized, or uncentralized neural network in the ancestral deuterostome. Yet, the anteroposterior ectodermal expression of 38 orthologous genes involved in chordate CNS patterning is nearly identical in hemichordates and chordates, although hemichordates have a diffuse neural net and chordates a centralized CNS [27]. Admittedly, major advances in neuroanatomy and function are often based on existing genes, but it is possible that the seemingly *de novo* appearance of PGBD5 in a basal cephalochordate may have been a functional innovation that contributed to subsequent evolution of the rudimentary lancelet CNS [59].

## Methods

### Human PGBD5 cDNA reconstructed

The human CX757968 EST clone, generated from a pluripotent male blastocyst cell line, was obtained from the I.M.A.G.E. Consortium (now distributed by Thermo Scientific, Pittsburgh, PA, USA). This EST is annotated as spanning PGBD5 exons 1–3, but upon resequencing we found it to be a 3′ partial mRNA spanning the last 43 residues of predicted exon 1, all of exons 2–7, and a 1779 nt 3′ UTR between the translation termination codon and poly(A) tail (new GenBank Accession No. KF670820). The missing 67 N-terminal residues downstream of a phylogenetically conserved initiator methionine were embedded in an exceedingly GC-rich CpG island which resisted genomic PCR. Instead, we reconstructed the 5′ end of the PGBD5 ORF *de novo* by Klenow extension and Pfu polymerase amplification of overlapping synthetic oligodeoxynucleotides [60]. The final PGBD5 cassette was a 2636 bp AscI fragment containing a 1575 bp PGBD5 ORF followed by a 1061 bp 3′ untranslated region; the cassette was cloned into a modified pET45b vector (pET45b-HA-Asc) for bacterial protein production.

### Search for additional PGBD5 orthologs or homologs

The complete human PGBD5 protein sequence encoded by exons 1–7 was used as a tBLASTn query to identify PGBD5 orthologs in the rat, mouse, zebra finch, chicken, frog, zebrafish, fugu, coelacanth, sea lamprey, and lancelet genomes. tBLASTn searches using the human, zebrafish, and lancelet PGBD5 proteins as queries were then carried out on the entire non-redundant nucleotide sequence collection (NCBI/NLM/NIH) as well as individual genome databases for 6 organisms: the sea squirt *Ciona intestinalis*, a urochordate (US Department of Energy, Joint Genome Institute); the sea urchin *Strongylocentrotus purpuratus*, an echinoderm (Human Genome Sequencing Center, Baylor College of Medicine); the acorn worm *Saccoglossus kowalevichii*, a hemichordate (Human Genome Sequencing Center, Baylor College of Medicine); *Hydra magnipapillata*, a Cnidarian (Center for Integrative Genomics, University of California at Berkele*y*); the sea hare *Aplysia californica,* a gastropod mollusk (Broad Institute through the UCSC Genome Browser); and the marine sponge *Amphimedon queenslandica,* a Poriferan (NCBI/NLM/NIH).

### Assembly of the sea lamprey and coelacanth PGBD5 sequences

A *Petromyzon marinus* (sea lamprey) PGBD5 sequence was initially found using the human PGBD5 query for a tBLASTn search of the March 2007 draft genome assembly (WUSTL v.3.0) generated by the Washington University Genome Sequencing Center and currently available on the UCSC Genome Browser. Contig9988 (with PGBD5 on the + strand) and contig34746 (with PGBD5 on the – strand) overlap, and each contig fills in large unsequenced tracts in the other. We found no hint of the conserved exon 1 although contig34746 has 2,565 bp upstream of exon 2; however, exon 1 is 68 kb upstream of exon 2 in humans, and might also be far upstream in lamprey. Sea lamprey has recently been found to undergo extensive DNA diminution during development, significantly complicating genome analysis [61-63]. The WUSTL v.3.0 lamprey assembly was subsequently recomputed and made available to us (JJ Smith, University of Kentucky, Lexington) in advance of publication [63]. The ortholog of human PGBD5 exons 2–7 was found in the 442 kb scaffold_316.1-442057 of the recomputed assembly, and annotated manually using the Ensembl predicted protein ENSPMAP00000004323 based on the WUSTL v.3.0 assembly. Although conserved exon 1 could not be found in the 204 kb of gapped sequence upstream of exon 2, it was ultimately located in a separate 4 kb scaffold_19254.1-4118. This small scaffold most likely maps within the large scaffold in 1 of 3 large unsequenced gaps (8, 29, and 63 kb) upstream of exon 2. PGBD5 exons 2–7 in the recomputed assembly differ only trivially from the Ensembl prediction based on the WUSTL assembly. The *Latimeria chalumnae* (coelacanth) ortholog of PGBD5 was kindly retrieved from contig_02766.1-262,491 of the draft genome assembly (CT Amemiya, Benaroya Research Institute and Department of Biology, University of Washington, Seattle) in advance of publication [64]. As was the case for lamprey, ORFs with homology to PGBD5 were identified and splice sites assigned manually. Coelacanth genome sequences have now been published by both the Amemiya [64] and Okada groups [65].

### Reconstruction of the lancelet PGBD5 gene

The complete lancelet PGBD5 gene spanning lancelet exons 1–7 is annotated as Protein and Transcript ID 79338 in the *Branchiostoma floridae* v1.0 genome assembly and was retrieved from the – strand of scaffold 84 generated by the US Department of Energy Joint Genome Institute (JGI) site (genome.jgi-psf.org/pages/search-for-genes.jsf?organism=Brafl1). The resulting lancelet PGBD5 protein sequence is based on predicted splice sites that (with one exception described below) maximize similarity to vertebrate PGBD5 homologs; the sole independent support for the predicted protein sequence is cDNA clone bflv024o04 encoding the N-terminal 83–86 residues of the lancelet protein (amphioxus.icob.sinica.edu.tw) [66]. Curiously, scaffold 84 has 2 × 60 bp imperfect tandem repeats overlapping exon 3, and 3 × 108 bp imperfect tandem repeats overlapping exon 4 (Additional file 1) whereas the equivalent lancelet genomic sequence on the UCSC Genome Browser (chrUn: 288364515–288370215) has 7 instead of 2 × 60 bp repeats and 6 instead of 3 × 108 bp repeats (Additional file 1). More curiously, as discussed below, the first of 2 tandem repeats overlapping exon 3 in scaffold 84 immediately abuts the 5′ splice site of exon 3, and the first of the 3 tandem repeats overlapping exon 4 immediately abuts the 5′ splice site of intron 4 (Figure 1, red underlines). Thus, neither the 2 × 60 bp repeats nor the 3 × 108 bp repeats would substantially affect the homology of lancelet with the lamprey and vertebrate proteins, were it not for the fact that JGI Protein and Transcript ID 79338 annotates almost all of the second and third 108 bp repeats as exon 5, potentially introducing an additional 57 residues into the lancelet protein with no apparent homology to the other PGBD5 proteins (Figure 1). As JGI exon 5 relies on predicted 5′ and 3′ splice sites without confirmation by EST or mRNA data, and the second of the 2 × 60 bp repeats is not translated, we decided to exclude JGI exon 5 from the lancelet PGBD5 protein, instead jump splicing exon 4 directly to exon 6. The resulting 13 residue deletion (Figure 1, red dashes) seems somewhat more plausible, both structurally and evolutionarily, than a 57 residue insertion in the midst of a highly conserved region of the PGBD5 protein.

Our attempts to eliminate the apparent 13 residue deletion, to reduce the predicted 57 residue insertion, or otherwise increase homology of lancelet with other PGBD5 sequences, either by use of alternative 5′ and 3′ splice sites, or by internal deletion of one or more imperfect tandem repeats in various registers, were unsuccessful. Drs Jia-Xing Yue and Nik Putnam (Rice University) graciously reexamined the original Sanger sequencing data [32] as well as new Illumina data generated since the original publication, but the new data independently confirmed the original sequence. Given unforced homology of lancelet PGBD5 with the lamprey and vertebrate proteins except in the vicinity of intron 4 and predicted exon 5, the imperfect 3 × 108 bp tandem triplication could reflect actual duplications, genetic heterozygosity, or even sequence assembly errors, although the re-sequencing data argue strongly against this last possibility.

### Lancelet PGBD5 genomic duplications include mRNA splice sites

The 60 and 108 bp tandem repeats that coincide with or overlap 5′ and 3′ splice sites echo earlier observations in lancelet: some lancelet globin genes have short tandem duplications of intron/exon boundaries (dubbed “mirages”) which apparently provide a mechanism for the creation of new introns within the genes [67]. Similarly, both the lancelet alcohol dehydrogenase (Adh) gene [68] and the troponin C (TnC) gene [69] contain “mirages” that duplicate splice sites yet do not generate aberrant mRNAs. The PGBD5 duplications are imperfect, unlike the globin, Adh, and TnC mirages, but all include intron/exon boundaries. These remarkable similarities suggest that tandem repeats in lancelet PGBD5 gene are real, but do not interfere with accurate mRNA splicing.

### Protein production

The PGBD5 cassette in the pET45b-HA-Asc vector generates recombinant PGBD5 (rPGBD5) with N-terminal hexahistidine and hemagglutinin tags and a predicted molecular mass of 65.9 kDa, about 7 kDa larger than the predicted 58.5 kDa for endogenous human PGBD5 protein. The rPGBD5 expression construct was transformed into Rosetta cells (Novagen EMD), induced with IPTG, and shaken overnight at room temperature. Cells were opened by sonication and cleared supernatants subjected to consecutive ammonium sulfate cuts of 0–25%, 25–40%, and 40–55% saturation. rPGBD5 was located in the 25–40% pellet by Western blotting, dialyzed, and affinity-purified over a Talon (cobalt) column followed by desalting over G25 Sephadex and further purification by ion exchange chromatography on CM-Sepharose. rPGBD5 protein purity was assessed by SDS-PAGE followed by Coomassie Brilliant Blue or silver staining.

### Antibody production

A rabbit polyclonal antibody against rPGBD5 was raised by R&R Research (Stanwood, WA, USA), affinity purified by binding to an NHS-Sepharose column coupled to rPGBD5, and eluted by low pH. The antibody immunoprecipitated human and mouse PGBD5 specifically, but cross-reacted with several abundant larger proteins after denaturation and immobilization on the polyvinylidene difluoride (PVDF) membrane for western blotting (Figures 7 and 8); the antibody also cross-reacted weakly with the hexahistidine tag but not with the HA tag on western blots (data not shown).

### Cell culture and transfection

The human HT1080 fibrosarcoma cells were grown in MEM alpha with 5% FBS and 1× penicillin/streptomycin. The human Burkitt’s lymphoma B lymphoblast Daudi cell line was grown in RPMI 1640 with 10% FBS and 1× penicillin/streptomycin. The *Trans*IT-LT1 reagent was used for transfections as recommended (Mirus Bio, Madison, WI, USA).

### Cell extracts

Daudi cells were washed once with PBS, resuspended in cold buffer D (20 mM Hepes pH 7.5, 50 mM NaCl, 2 mM MgCl_2_, 5% glycerol) and lysed by incubation with 0.1% NP-40 for 10 min on ice. Nuclei were separated from cytoplasm by centrifugation for 5 min at 13,000 rpm in a microfuge. An equal volume of 4× SDS-PAGE loading buffer was added and the sample sonicated to reduce viscosity.

### Mouse brain extracts

Mouse whole brain extracts were initially prepared from fresh BALB/c brain by Dounce homogenization in buffer D containing 1 mM PMSF, 0.5X protease inhibitor cocktail (Roche) and sonicated to shear genomic DNA. Subsequently, cell fractions were prepared from flash frozen brains of 8–10 week old Swiss Webster mice (Pel-Freez Biologicals, Rodgers, AR, USA). The brain was thawed on ice, diced with a sterile scalpel, and homogenized by 25 strokes with the loose pestle of a 5 mL Dounce in 5 mL of buffer D. The resulting emulsion was sonicated to generate whole brain homogenates, or centrifuged for 20 min at 10,000 rpm in a Beckman JA-21 rotor to separate cytoplasm and nuclei. The crude nuclear pellet was washed with an equal volume of buffer D + 1 mM PMSF, respun, and then resuspended in 2.5 mL of buffer D + 10% glycerol + 1 mM PMSF for storage at −70°C. The fractionation and integrity of nuclei throughout the preparation were monitored by air drying an aliquot on a glass cover slip. The cover slips were immersed in PBS+DAPI, washed briefly in PBS, and inspected under the microscope for intact fluorescent nuclei. To determine whether PGBD5 binds to chromatin or DNA, crude nuclei were repeatedly sonicated in 10 sec bursts, assaying each time by DAPI staining until all nuclei were broken. Nuclear aliquots were treated with 0.1 U/μL freshly prepared DNAse I. The desired final concentration of NaCl was then added to DNase I treated and untreated nuclei, and the aliquots incubated for 15 min at room temperature before centrifugation to separate supernatant from the salt insoluble pellet. To prepare pure nuclei, crude nuclei were pelleted, resuspended in buffer D + 1 M sucrose, layered over a 1.8M sucrose cushion buffer D, and centrifuged for 1 h at 22,000 rpm (32,000 × *g*) in an Beckman Optima tabletop ultracentrifuge. The pelleted nuclei were resuspended in buffer D + 0.32 M sucrose and assayed by DAPI staining and western blotting. Mouse brain extracts were immunoprecipitated using anti-rPGBD5 antibody and the Thermo Pierce Direct IP kit for SDS PAGE and western blotting.

## Abbreviations

CNS: Central nervous system; CSB: Cockayne syndrome Group B; EST: Expressed sequence tag; ORF: Open reading frame; PAGE: Polyacrylamide gel electrophoresis; PGBD: PiggyBac domain; rPGBD5: Recombinant PGBD5; TF: Transcription factor.

## Competing interests

The authors declare that they have no competing interests.

## Authors’ contributions

TP, LTG, SLP, ADB, and AMW conceived, designed, and performed the experiments. TP, SLP, and AMW analyzed the data and wrote the paper. All authors approved the final manuscript.

## Supplementary Material

Additional file 1 PDF file showing that the available lancelet PGBD5 genomic sequences are complicated by tandem duplications within presumed protein coding sequences. Click here for file

Additional file 2 Intron positions do not suggest PGBD5 ancestors. Click here for file

Additional file 3 **Cladogram of PGBD5 sequences shown in Figure **1** using human PGBD1, 2, 3, and 4 as an outgroup.** Click here for file

Additional file 4 Tissue of origin of vertebrate PGBD5 mRNAs and spliced ESTs. Click here for file

Additional file 5 Multiple clusters of occupied transcription factor binding sites in the human PGBD5 locus. Click here for file
