# Supplementary material for: PGBD5: a neural-specific intron-containing piggyBac transposase domesticated over 500 million years ago and conserved from cephalochordates to humans
Source: Mob DNA. 2013 Nov 1;4:23. doi: 10.1186/1759-8753-4-23 (PMC3902484; doi:10.1186/1759-8753-4-23)
Supplement: Additional file 1 — PDF file showing that the available lancelet PGBD5 genomic sequences are complicated by tandem duplications within presumed protein coding sequences. [file 1759-8753-4-23-S1.pdf]

Additional file 1. The available lancelet PGBD5 genomic sequences are complicated by tandem duplications within presumed protein coding sequences. The apparently complete lancelet PGBD5 gene spanning exons 1-7 is annotated as Protein and Transcript ID 79338 in the *B. floridae* v1.0 genome assembly and was retrieved from the (–) strand of scaffold 84 (http://genome.jgi-psf.org/pages/search-for-genes.jsf?organism=Brafl1). The 7 lancelet coding exons are shown in red font; for illustrative purposes, exon 1 begins with the AUG initiator and exon 7 ends with UAG terminator because the transcription start site and polyadenylation site cannot be reliably predicted. Introns as well as DNA flanking the 5' and 3' ends of the ORF are shown in black. For clarity, each set of imperfect tandem repeats is highlighted alternately in yellow and cyan. Note that the XM_002604443 mRNA model encodes a more divergent protein sequence than the one shown in Figure 1 because the imperfect tandem repeats differ in the corresponding genomic sequence on the UCSC Genome Browser (chrUn: 288364515-288370215) which has 7 x 60 bp repeats instead of 2 overlapping exon 3, and 6 x 108 bp repeats instead of 3 flanking and including exon 5.

```
>scaffold84
GACCTTTGACATGTAGTTCTAAACTGTGTGGCAAGTGAGAGGCAAGAAGAAATTGTTTGCTGTAATAAATTTTACAGTGTTTCTGAAGGTATATAGAGCC 847700
TCACATTGACTTAAATCTGCAGAATATCCTGTAGCTGACCTTAGCATAGTCAGCATCTCAGGAAGTGCCCCGCCCTCAATCCCCCTGCAGCATCAGCACC 847600
ATGGATGATGAGGAGGAGCAGAGACGGCCGGCCGGCCTCCAACATCTCCTGAGACCTCCGTCCAACCCGGCGGATCACGAGGGGCTCCAGATTTCCGACG 847500 ex1
ACATGTTCGGCGGGTCGGCAAGCTCGGACGACGCCAGCACCGGCTCCGAGATGAGCCAATCGGATGATGATGATGAGGATTCTGAGGAGATGGGTGATGC 847400
AGATGAGGATGGAGGCGGCAGTGCCGATGATGGAGGGTGGACGGCCGACATCACTCACTTCGACCCCCCGTGCTTCAGCGACGGCGAAACGGTCGGGCCG 847300
GTCCGAGACGTCACGCCGGACATGCGGCCGATCGACTTCTTCCACCTGCTGGTCCCGGAGCCCGTCCTGAGCGCCATCGTGCAGCAGACCAATCGCTATG 847200
CACACAAGTACCAGCTGAAGAACGGCCGGGACGAGGAGTGGCGGAATGTGGACCTCGCGGAGATGAAGGTATTGATTGAAAAGACTCATTAGGACTTTAA 847100
ACTGTATCTCTACATTAATGCGTTGTCTGACCCTTGCCTCTTCCCTCATGACCTCAATGAAAAGTAGCCTACGGGCCGATTTGAGCTTTCATGAAAAAAC 847000
AATGGTTCAAACAAATTATGCTCCAGGTTGTGTCCTGATGCTTATTTGTGAGGCTGTGTCTTTTAATTACTTTTGTCCGTCCCACTCATTGTTTGGGACC 846900
ATCATGCATGTTGTTAATTTTCCATGTTAATCTTTCCCAATTCCATTTTAAACTTAGGAAAGCACATGTACCGTAGTTACATGTCAGGAAGTGAAGATAT 846800
TTTGGACAGATGGGGTGGAATTTTTTTTCCGTCCCAACAGGCAAATTTCTGTCCCAGGACGGAGTGACAGGTCCTGGAGATGGTCCTGTGCAGCAGCATA 846700
TTTAAGGGGGGCTGAACTGGAAATTGAAAAGCCCACTACACATACAGTAGTACATTATGCTCAGATATAGCCCCAAAGTCACAAAATACCACAAATTATT 846600
TATGGGTGAAATCTTGTCCATTTCGCAGGCGTTCATGGCCATCTTCATGTACACCAGCCTGGTGCACGGCGTCTTCTCCGAGGACATCTCCGCCCTGTGG 846500 ex2
GAGGCTGACGAGATAACGTACAACCCGGTCATGGCGAGCATCCTGACGCAGCGCCGCTTCCAGCGCATCATGCGCTACTTCCACGTGGTGTCCTCCAGCG 846400
GCCAGCAGCAGGGCGACAGTCTGCGCAAGATCCGCCCCTTCCTCGAGACCGTGCAGAACTCCTTCCGGAATGAGTTCAAGACTGCCCAGGTACCTGATAC 846300
TGCAATTCCTGATTTTTCCAATGTACTTTTATGTTGAATGGTTTTCGGTTTTCATGGTGACGACTGTAACGAGAACTTATCATTACTGATAACAAATAAT 846200
TTGTCTTGGTGACAGTAAGAAATGATTCGTTCCCATGAACATTTAGTTCCAAAAAGTTAAATTTTCTTTTTTAGCATATCTTATTACCTGGATGTCTAAT 846100
CTTCATTGACGAATAACAAAGGTATTTTGACATCTTCGCTTTAGACCCACGTGATCCACGAGCCTCCGCTGGAGGAGGAGCCGCTGATGTGGTCGCGCCA 846000 ex3
CCTGGACAGGAAACGGCGCAAGAAGAAACGGTTCAACCTCTGGGTGCGACAGTGCACAGCTACAGGGTTCATCTCCCAGGTGAGCTGTCTGTGTGTTCCT 845900
CCATGTTTACAGTGCACAGCTACAGGGTTCATCTCCCAGGTGAGCTGTCTGTGTGTTCCTCCATGTTTGATGTCCATAACCCTATCTAGGGGTGGGTACC 845800
TAAGTACAAGGTAAAATGGTCTCATTCCTGTATTTGGCCGTCCCATTGGAATATACACTTTCTATGCCAAAAAATTCCATTAAGACAGTATATCTGGAGA 845700
TGTTTTGCTTTCTATCGCAAGCACAGCCGTTAATGTTACATGTAGGAAATAAAAAAATTGCCACCTAAATCCAGCGCAATCCAAAATGCTGGAGTGGCTC 845600
CACGAGCCTTGTTTTGAAGTGAATCCCCCCTAGTAAATAAAAAAAAAATAAACATGTATCTGCTTTCCTAGATCCACATCCATGTGAAGGAGAAGAGCGA 845500 ex4
CAGCCTGAGCCACCACGAGAAAGGTTACTCCGTCGCCCGCGACCTCTGCTCCGACCTGATTGGCTCGGGACGGAACCACATTGTGTTCTGCATCCCGGCC 845400
AACACCAGCCTGCGTCTGTTCCGCGAGCTCGAGTCCCACGGCGTGTACGGCTGCGGGGTCGTCACGGACGCCAGGCACACCGACTGCGAGGGACTCCCTG 845300
CCAGCGTTCTCGCCAAGCCTCCGACATCCCTGCGCAAGTATGACAGCATCGTCCGCACCAAGGGCAGGTTTGTTTGTTTGTTTGTTTGTTTGTTTACTAG 845200
ATGGTATCCCCGCCAGCGTTGTCGCCAAGCCTCCGGCATCCCTGCGCAAGTATGACAGCGTCGTCCGCACCAAGGGCAGGTTTGTTTGTTTGTTTGTTTG 845100 ex5
TTTACTAGATGGTATCCCCACCAGCGTTCTCGCCAAGCCTCCGGCATCCCTGCGGAAGTATGACAGCATCGTCCGCACCAAGGGCAGGTTTGTTTGTTTG 845000
TTTGTTTGTTTACTTGCTTAGAATCTCCAGTAAAGTACTCATAAAAATACATCATGCTCTTAGTAGTCTTGCTTGAGACAATTTAACACATGACTCTACA 844900
GCTTACTGAAGCTTCACTATTATATGCAAACCTAGCAATAACAACTGAGATAGAAATGTCACAGAGAGAGCAGAGGTCTGAATTGTTAGTTGATGTGACA 844800
CCTGGTAGTTTTTGCGTACCATGTACATAGTGTCTACTTTTGTCCATTTCTTGATACTTTGTTCCAATGCAGATATTCCGCTGTGTTGTGGAACTCGCCA 844700
CGCAACCCCTTCAGGTTTGTCTGTTCATTTGTTTCAAACAACAAGTTACTAAGGTTAAACCCCCTGTAAACAACTGTAAATAAAGTACAAGCTGCATATC 844600
CAGTCAGGATTATTTCATCTTCTCACACAGAAATGGACCATTTTCGATAAAGTATGGTGGATTCTTTAACATGCTCTAGGTGTGGCTCTCTTTAAACATG 844500
GGGCCTCCAGATTTATGTCCTATCAAAGCTAGGCACTCGATTTTACCTGACCCAAGTGATGAAGTAGGTGTAAAGTTCTATTTAGAAAGGCACGACTTTG 844400
GGGTCTGGTGGAGTTTAAACCCAGAACCAGTAGATTCCAAGTCAATATCAAACATTCGCGGGCATCATAGTTCAAGTCATCAAGTCAGTAGCCTGACTAA 844300
AATCACACTCCTAGTTGCCACCACCTAGGAGCAGGTCATTAACCCCAGCCAGCAAGAGACTGTGTGAATACAGGCTAACAATTGTCAACAACTCTCACAA 844200
CAAGACCACTTTGCCCCCCTCCCCCCAGGTTGCTGACGAACGCGTTCTCGCCCGCGGCGCAGGGGAAGCTGGTCGTGAAGAAGAACCGGAGGCCTGACGA 844100 ex6
GGAACGGCCGTGTCTCGAGGCTGTGCAGGCCTTCCAGGTGGGCTGCTTCACTTTGTTTGTTTGTTTGTTTGCTTGTTTGTTTTTATTGCATACACAGTAA 844000
AATTCCCTCATGGTGTAACACAATGTGGTCCCAAATGAATATAGGCATGTGATTATTATGATACTGTAAAATGCATGTAGATTCGCAGGGATTTAATTTC 843900
GTGGTAGCGGGAAAAAGGACTTTTTGCGGTGGTATTAAGTTCGCAGTAGCACCTGAGGCACTGTACTCTCTTACTGTTACTGCCATGGAAAAATGTTCGC 843800
GGTGGTTTTAAGGTTGCGGTGAAGGGACCACCGCAAAAACCGCGAACATTAAACCACCGCGAACGTTTCTGCATTTACAGTATATTCTTGACAATAGTGA 843700
TGACAATACAACAATGTACATCTACTTCTTAAGAATCTTCTCCTCTGTTTCAGAACCACATCAAGTACATCGACAAGTACAACGAGAAGTACTCTTGGTA 843600 ex7
CATCATCTCCTACAAGCCCAGCAAGACGTGGCAGCAGCTGTTCTGGACGGTCCTCAGCCTGGCCATGAACAACGCCTACATCATGTACAAGCTGTCCCCG 843500
GCGCACGCGCACTCCCGTCTCAGCCGCAGCGACTTCGGGGCAGAGCTGCTGCGCGCCATGGCCGGGCCGTACTACCAGGAGCCCCCCACGCAGGGGGAGG 843400
AGTAGCCCCCCACCCAGGGGGAGGAGTAGTGCGTGCCATGGCCAGGCCGTACTACCAGGAGCCCCCCACCAAGGGGGAGGAGTAGTGCGGGCCATGGCCA 843300
GGCCGTACTACCAGGAGCCCCCCACCCAAGGGGAGGGGTAGTGCGCGCCATGGCTGGGCCGTACTACCAGGAGCCCCCCACCCAGGGGGAGGAGTAGTGC 843200
GCGCC                                                                                                843195
```
