# Supplementary material for: PGBD5: a neural-specific intron-containing piggyBac transposase domesticated over 500 million years ago and conserved from cephalochordates to humans
Source: Mob DNA. 2013 Nov 1;4:23. doi: 10.1186/1759-8753-4-23 (PMC3902484; doi:10.1186/1759-8753-4-23)
Supplement: Additional file 2 — Intron positions do not suggest PGBD5 ancestors. [file 1759-8753-4-23-S2.pdf]

Additional file 2. Intron positions do not suggest PGBD5 ancestors.

We constructed a cladogram of selected piggyBac elements including Human PGBDs (Hs-PGBD1, 2, 3, 4, 5); the Tetrahymena (Tt-TPB2) and Paramecium (Pt-PGM) piggyMacs; the *Myotis* piggyBat (pBat-Ec); and the cabbage looper moth piggyBac (Tni-PGBD). Complete protein sequences were aligned using EBI Clustal Omega. The homologous transposase regions were then realigned and used to generate a Neighbor-joining tree without distance corrections. Distances are given in parentheses. In an exhaustive phylogenetic tree, Sarkar et al. (2003) also clustered PGBD1, 2, and 3, noting that PGBD4 and 5 were "quite distinct" and that PGBD5 was the "most highly divergent" [1].

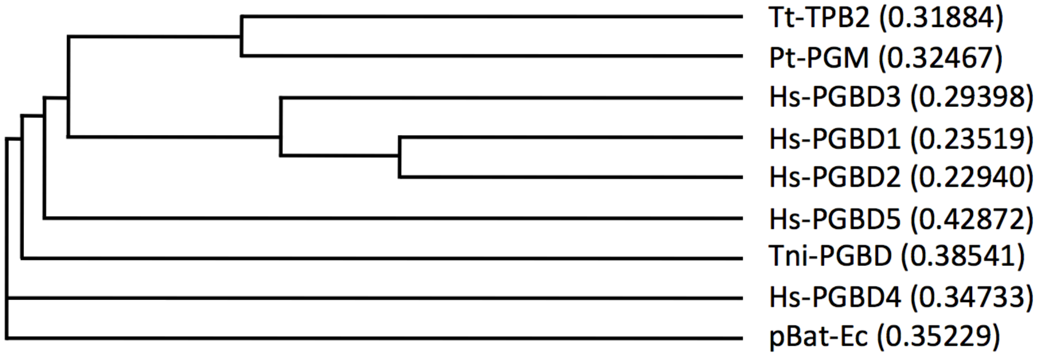

Of the many thousands of sequenced piggyBac elements, only 3 are known to have introns within the transposase ORF: the vertebrate PGBD5s, and the 2 active ciliate piggyMacs. The transposase domains of the two active ciliate piggyMacs cluster with human PGBD1, 2, and 3 but are only slightly more related to each other than to the human piggyBacs; the *Trichoplusia ni* (cabbage looper moth) piggyBac [2] and *Myotis lucifugus* (little brown bat) piggyBat [3] transposases cluster with human PGBD4; and human PGBD5 does not group with any of the others (see cladogram below). In addition, the *Paramecium tetraurelia* PGM piggyMac homolog has 2 very small introns (paramecium.cgm.cnrs-gif.fr) [4] conforming to the stringent length (20-34 nt) and splice site constraints typical of other *P. tetraurelia* introns [5], but neither intron aligns with any of the 12 canonical introns (47-153 nt) in the *Tetrahymena thermophila* TPB2 piggyMac homolog (broadinstitute.org/annotation/genome/Tetrahymena) [6]. As a result, it is not surprising that a comparison of PGBD5 intron positions in lancelet, lamprey, and vertebrates (Figure 1) with those in the two ciliate piggyMacs suggests that the ciliate introns are unlikely to align with any of the introns in the chordate or vertebrate lineage, even after making generous allowance for intron sliding. Thus, intron positions do not suggest ancestors, relatives, or progeny of the PGBD5 lineage that first appears in lancelet.

References

1. Sarkar A, Sim C, Hong YS, Hogan JR, Fraser MJ, Robertson HM, Collins FH: **Molecular evolutionary analysis of the widespread piggyBac transposon family and related "domesticated" sequences.** *Mol Genet Genomics* 2003, **270:**173-180.
2. Handler AM, McCombs SD, Fraser MJ, Saul SH: **The lepidopteran transposon vector, piggyBac, mediates germ-line transformation in the Mediterranean fruit fly.** *Proc Natl Acad Sci U S A* 1998, **95:**7520-7525.
3. Mitra R, Li X, Kapusta A, Mayhew D, Mitra RD, Feschotte C, Craig NL: **Functional characterization of piggyBat from the bat Myotis lucifugus unveils an active mammalian DNA transposon.** *Proc Natl Acad Sci U S A* 2013, **110:**234-239.

4. Baudry C, Malinsky S, Restituito M, Kapusta A, Rosa S, Meyer E, Betermier M: **PiggyMac, a domesticated piggyBac transposase involved in programmed genome rearrangements in the ciliate Paramecium tetraurelia.** *Genes Dev* 2009, **23:**2478-2483.
5. Jaillon O, Bouhouche K, Gout JF, Aury JM, Noel B, Saudemont B, Nowacki M, Serrano V, Porcel BM, Segurens B, et al: **Translational control of intron splicing in eukaryotes.** *Nature* 2008, **451:**359-362.
6. Cheng CY, Vogt A, Mochizuki K, Yao MC: **A domesticated piggyBac transposase plays key roles in heterochromatin dynamics and DNA cleavage during programmed DNA deletion in Tetrahymena thermophila.** *Mol Biol Cell* 2010, **21:**1753-1762.
