# Supplementary material for: PGBD5: a neural-specific intron-containing piggyBac transposase domesticated over 500 million years ago and conserved from cephalochordates to humans
Source: Mob DNA. 2013 Nov 1;4:23. doi: 10.1186/1759-8753-4-23 (PMC3902484; doi:10.1186/1759-8753-4-23)

Additional file 3. Cladogram of PGBD5 sequences shown in Figure 1 using human PGBD1, 2, 3, and 4 as an outgroup.

We constructed a cladogram of all PGBD5 proteins shown in Figure 1, with the human PGBD1, 2, 3, and 4 core sequences as an outgroup, using EBI Clustal Omega to generate a Neighbor-joining tree without distance corrections. Distances are given in parentheses. We defined PGBD1, 2, 3, and 4 "core" sequences as including all detectable similarity between the complete RefSeq protein sequences but excluding unalignable N- and C-terminal regions.

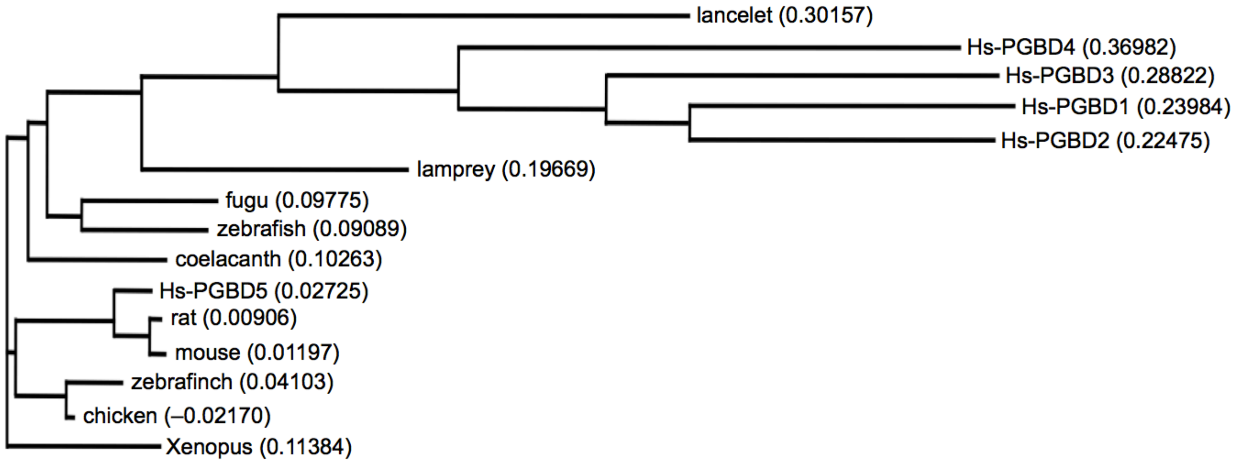

Supplement: Additional file 3 — Cladogram of PGBD5 sequences shown in Figure 1 using human PGBD1, 2, 3, and 4 as an outgroup. [file 1759-8753-4-23-S3.pdf]
