# Supplementary material for: PGBD5: a neural-specific intron-containing piggyBac transposase domesticated over 500 million years ago and conserved from cephalochordates to humans
Source: Mob DNA. 2013 Nov 1;4:23. doi: 10.1186/1759-8753-4-23 (PMC3902484; doi:10.1186/1759-8753-4-23)
Supplement: Additional file 4 — Tissue of origin of vertebrate PGBD5 mRNAs and spliced ESTs. [file 1759-8753-4-23-S4.pdf]

Additional file 4. Tissue of origin of vertebrate PGBD5 mRNAs and spliced ESTs.

We include all UCSC Browser mRNAs but only spliced ESTs spanning 3 or more documented exons. Tissues are adult or unknown except where noted otherwise. Only human, mouse, and Xenopus had more than 1 or 2 mRNAs or spliced ESTs, and zebrafish had none.

Accession	organism	EST or mRNA	tissue of origin
BC036865	human	mRNA	brains, pooled
BC027466	human	mRNA	brain, anaplastic oligodendroglioma
AL833243	human	mRNA	brain, amygdala
AK021475	human	mRNA	whole embryo, "mainly head"
BC013901	human	mRNA	lung, small cell carcinoma
AK297321	human	mRNA	brain, fetal
AK315968	human	mRNA	brain, amygdala
BC150638	human	mRNA	11 pooled tissues including cerebellum
BE798312	human	EST	lung, small cell carinoma
DA283023	human	EST	brain, corpus callosum
BX420971	human	EST	brain, fetal
DC350986	human	EST	brain, fetal
CN302362	human	EST	embryonic stem cell
DC315602	human	EST	brain, amygdala
DN992101	human	EST	brain
DC411558	human	EST	kidney, tumor
BI757418	human	EST	unknown
BU186080	human	EST	retinoblastoma
CX757968	human	EST	pluripotent blastocyst inner cell mass
BC094384	mouse	mRNA	eye
BU703358	mouse	EST	brain, embryo
BY248314	mouse	EST	brain, visual cortex
CO424744	mouse	EST	eye, newborn
CK621322	mouse	EST	retina
BY245073	mouse	EST	brain, visual cortex
CA316156	mouse	EST	brain, embryo
CF724891	mouse	EST	eye, embryo
CF724891	mouse	EST	eye, embryo
CX850194	Xenopus	EST	brain
DT415449	Xenopus	EST	oviduct
BX723479	Xenopus	EST	tadpole
CX440952	Xenopus	EST	gastrula
AL880779	Xenopus	EST	egg
CX331004	Xenopus	EST	tadpole
AL882498	Xenopus	EST	egg
DT399770	Xenopus	EST	oviduct
DT399770	Xenopus	EST	oviduct
