# Supplementary material for: PGBD5: a neural-specific intron-containing piggyBac transposase domesticated over 500 million years ago and conserved from cephalochordates to humans
Source: Mob DNA. 2013 Nov 1;4:23. doi: 10.1186/1759-8753-4-23 (PMC3902484; doi:10.1186/1759-8753-4-23)
Supplement: Additional file 5 — Multiple clusters of occupied transcription factor binding sites in the human PGBD5 locus. [file 1759-8753-4-23-S5.pdf]

Additional file 5. Multiple clusters of occupied transcription factor binding sites in the human PGBD5 locus. For simplicity, this modified screen capture displays only the most highly occupied transcription factor binding sites (TFBSs) seen in 62 nonneural cell lines and 1 cerebral neuroectodermal tumor (PFSK-1) used to build the current ENCODE TF ChIP-seq database available for hg19 on the UCSC Genome Browser.

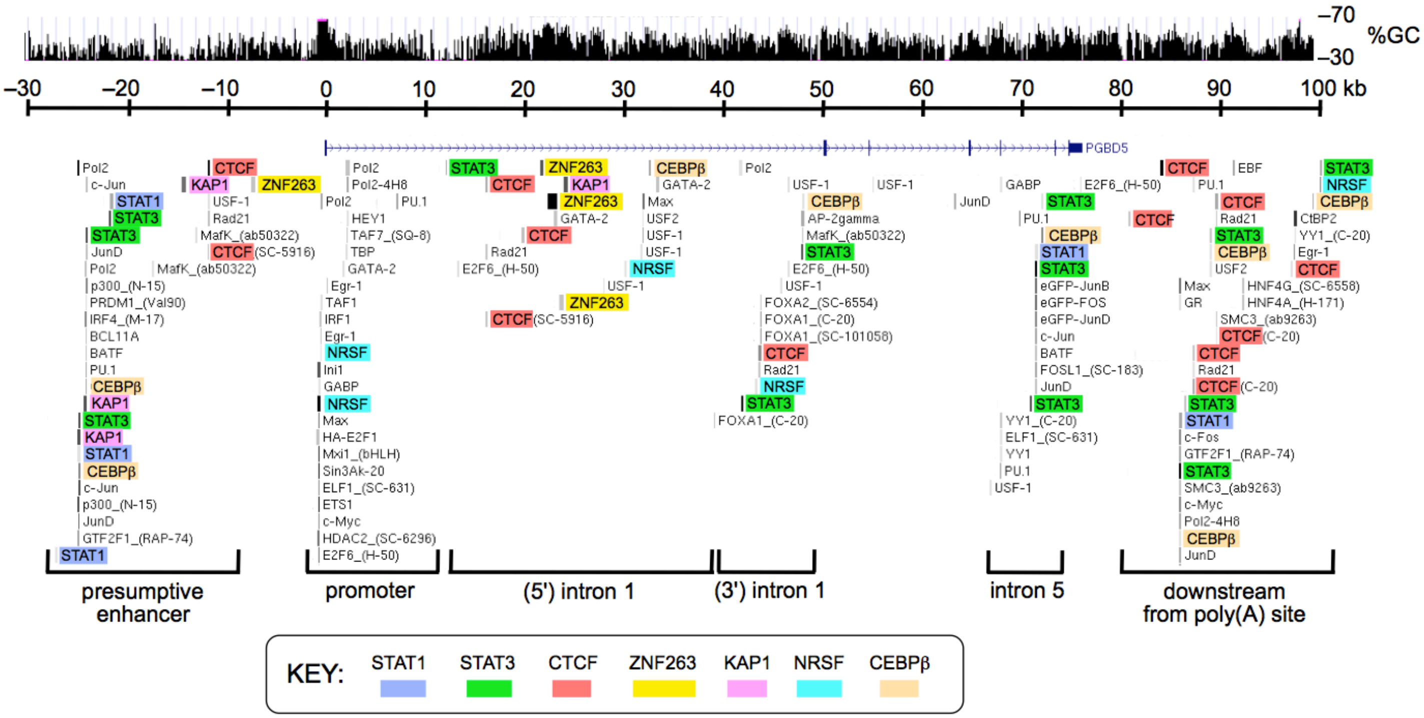

The 130 kb PGBD5 neighborhood appears to be enriched in sites occupied by STAT3 (13 sites), CTCF (11 sites), and CEBPβ (8 sites):

STAT3 binds a core set of genes that control STAT3 functions and cell growth, but also collaborates with other TFs to drive cell type-specific transcription programs [1]. In addition to suppressing neurogenesis while activating astrogliogenesis in cultured neural stem cells [2-4], STAT3 can form a potent repressive complex with the SIN3A [5] and KAP1 corepresssors [6, 7], as well as with DMNT1 (DNA methyltransferase 1) and HDACs [5, 8, 9].

CTCF is a sequence-specific DNA binding factor responsible for chromatin silencing, activation, and looping [10]. As for STAT3, the occupied CTCF sites are distributed from upstream of the promoter to downstream of the polyadenylation site. Although CTCF-induced DNA looping often silences chromatin domains, it can also activate promoters [11] possibly by strengthening TF binding to low affinity motifs or when CTCF is evicted by inducible transcription of noncoding RNA [10].

None of the cells in the TF ChIP-seq datasets is neuronal, but there appear to be 6 distinct clusters of occupied TF binding sites (denoted by brackets). In fact, clustered TF binding sites (TFBSs) are a well known indication of bona fide regulatory regions in organisms ranging from metazoans [12] to mammals [13]. Interestingly, 5 of the 6 clusters contain binding sites for CEBPβ (CCAAT/enhancer-binding protein β), a neural TF with diverse functions in neural differentiation, development, learning, and memory [14]. The enhancer and promoter TFBS clusters are undoubtedly real, but any of the other 4 clusters could reflect a combination of GC-richness, nucleosome-depletion, DNase I sensitivity, and well-positioned flanking nucleosomes that favors TF binding [15].

Many ultraconserved elements (UCEs) are conserved from fish to humans [16] and about 30% of all UCEs tested have been found to serve as enhancers in the developing central and peripheral nervous system [17]. However, using the "Ultra" custom track of the UCSC Genome Browser [16], data last updated 2013-05-18), the UCEs nearest human PGBD5 were a distant 13 Mbp upstream and 15 Mbp downstream (data not shown).

References

1. Hutchins AP, Diez D, Takahashi Y, Ahmad S, Jauch R, Tremblay ML, Miranda-Saavedra D: **Distinct transcriptional regulatory modules underlie STAT3's cell type-independent and cell type-specific functions.** *Nucleic Acids Res* 2013, **41:**2155-2170.
2. Gu F, Hata R, Ma YJ, Tanaka J, Mitsuda N, Kumon Y, Hanakawa Y, Hashimoto K, Nakajima K, Sakanaka M: **Suppression of Stat3 promotes neurogenesis in cultured neural stem cells.** *J Neurosci Res* 2005, **81:**163-171.
3. He F, Ge W, Martinowich K, Becker-Catania S, Coskun V, Zhu W, Wu H, Castro D, Guillemot F, Fan G, et al: **A positive autoregulatory loop of Jak-STAT signaling controls the onset of astrogliogenesis.** *Nat Neurosci* 2005, **8:**616-625.
4. Coskun V, Zhao J, Sun YE: **Neurons or glia? Can SHP2 know it all?** *Sci STKE* 2007, **2007:**pe58.
5. Icardi L, Mori R, Gesellchen V, Eyckerman S, De Cauwer L, Verhelst J, Vercauteren K, Saelens X, Meuleman P, Leroux-Roels G, et al: **The Sin3a repressor complex is a master regulator of STAT transcriptional activity.** *Proc Natl Acad Sci U S A* 2012, **109:**12058-12063.
6. Tsuruma R, Ohbayashi N, Kamitani S, Ikeda O, Sato N, Muromoto R, Sekine Y, Oritani K, Matsuda T: **Physical and functional interactions between STAT3 and KAP1.** *Oncogene* 2008, **27:**3054-3059.
7. Jakobsson J, Cordero MI, Bisaz R, Groner AC, Busskamp V, Bensadoun JC, Cammas F, Losson R, Mansuy IM, Sandi C, Trono D: **KAP1-mediated epigenetic repression in the forebrain modulates behavioral vulnerability to stress.** *Neuron* 2008, **60:**818-831.
8. Zhang Q, Wang HY, Marzec M, Raghunath PN, Nagasawa T, Wasik MA: **STAT3- and DNA methyltransferase 1-mediated epigenetic silencing of SHP-1 tyrosine phosphatase tumor suppressor gene in malignant T lymphocytes.** *Proc Natl Acad Sci U S A* 2005, **102:**6948-6953.
9. Lee H, Zhang P, Herrmann A, Yang C, Xin H, Wang Z, Hoon DS, Forman SJ, Jove R, Riggs AD, Yu H: **Acetylated STAT3 is crucial for methylation of tumor-suppressor gene promoters and inhibition by resveratrol results in demethylation.** *Proc Natl Acad Sci U S A* 2012, **109:**7765-7769.
10. Merkenschlager M, Odom DT: **CTCF and cohesin: linking gene regulatory elements with their targets.** *Cell* 2013, **152:**1285-1297.
11. Guo Y, Monahan K, Wu H, Gertz J, Varley KE, Li W, Myers RM, Maniatis T, Wu Q: **CTCF/cohesin-mediated DNA looping is required for protocadherin alpha promoter choice.** *Proc Natl Acad Sci U S A* 2012, **109:**21081-21086.
12. Berman BP, Nibu Y, Pfeiffer BD, Tomancak P, Celniker SE, Levine M, Rubin GM, Eisen MB: **Exploiting transcription factor binding site clustering to identify cis-regulatory modules involved in pattern formation in the Drosophila genome.** *Proc Natl Acad Sci U S A* 2002, **99:**757-762.
13. Bolouri H, Ruzzo WL: **Integration of 198 ChIP-seq datasets reveals human cis-regulatory regions.** *J Comput Biol* 2012, **19:**989-997.
14. Kfoury N, Kapatos G: **Identification of neuronal target genes for CCAAT/enhancer binding proteins.** *Mol Cell Neurosci* 2009, **40:**313-327.
15. Wang J, Zhuang J, Iyer S, Lin X, Whitfield TW, Greven MC, Pierce BG, Dong X, Kundaje A, Cheng Y, et al: **Sequence features and chromatin structure around the genomic regions bound by 119 human transcription factors.** *Genome Res* 2012, **22:**1798-1812.
16. Bejerano G, Pheasant M, Makunin I, Stephen S, Kent WJ, Mattick JS, Haussler D: **Ultraconserved elements in the human genome.** *Science* 2004, **304:**1321-1325.
17. Pennacchio LA, Ahituv N, Moses AM, Prabhakar S, Nobrega MA, Shoukry M, Minovitsky S, Dubchak I, Holt A, Lewis KD, et al: **In vivo enhancer analysis of human conserved non-coding sequences.** *Nature* 2006, **444:**499-502.
